# Assessing the effect of β-glucan diets on innate immune response of tilapia macrophages against trichlorfon exposure: an in vitro study

**DOI:** 10.1007/s10695-023-01283-5

**Published:** 2023-12-15

**Authors:** Camino Fierro-Castro, Lluís Tort, Fábio Erminio Mingatto, Jaqueline Dalbello Biller

**Affiliations:** 1 Departamento de Biología Molecular, Área de Genética, Facultad de Ciencias Biológicas y Ambientales, Universidad de León, 24071 León, Spain; 2 Departmento de Biología Celular, Fisiología E Inmunología, Fac. Biociencas, Universitat Autònoma de Barcelona, 08193 Bellaterra, Spain; 3 Department of Animal Science, College of Agricultural and Technological Sciences, São Paulo State University (Unesp), Dracena, Brazil

**Keywords:** Aquatic contaminants, Trichlorfon, Organophosphates, Innate immunity, Immunomodulation, Macrophages, *Oreochromis niloticus*

## Abstract

The widespread use of pesticides in some areas where fish species such as tilapia are farmed may cause damage to the environment and affect commercial fish and therefore, human health. Water leaching with the pesticide trichlorfon, during the fumigation season in the field, can affect water quality in fish farms and consequently affect fish health. At the same time, the use of immunomodulatory compounds such as β-glucan supplied in the diet has become widespread in fish farms as it has been shown that improves the overall immune response. The present research examines the immunomodulatory impacts observed in macrophages of Nile tilapia (*Oreochromis niloticus*) after being fed a diet supplemented with β-glucan for 15 days, followed by their in vitro exposure to trichlorfon, an organophosphate pesticide, at concentrations of 100 and 500 µg mL^−1^ for 24 h. The results showed that β-glucan diet improved the viability of cells exposed to trichlorfon and their antioxidant capacity. However, β-glucan did not counteract the effects of the pesticide as for the ability to protect against bacterial infection. From the present results, it can be concluded that β-glucan feeding exerted a protective role against oxidative damage in cells, but it was not enough to reduce the deleterious effects of trichlorfon on the microbicidal capacity of macrophages exposed to this pesticide.

**Supplementary Information:**

The online version contains supplementary material available at 10.1007/s10695-023-01283-5.

## Highlights

- The B-glucan diet enhances cell viability when exposed to trichlorfon and boosts their antioxidant capacity.
- Trichlorfon exposure triggers multiple immune responses, including overexpression of immune-related genes and intracellular ROS production in macrophages.
- Prior consumption of B-glucan reduces ROS production, exerting a protective role against cellular damage in these cells.
- The pesticide's impact diminishes the cell's ability to defend against bacterial infections.

**Supplementary Information:**

The online version contains supplementary material available at 10.1007/s10695-023-01283-5.

## Introduction

Nile tilapia, *Oreochromis niloticus*, is one of the most cultured commercial fish species, for which China, Thailand, and Brazil are among the largest producers. Nile tilapia was introduced to developing countries and farmed in order to meet local requirements for protein intake. By improving production techniques and product flavor control, the protein requirements for human consumption improved, which favored the entry of tilapia into the main fish markets in those countries (FAO 2021). Tilapias are fast-growing fish, easy to breed and farm intensively, and relatively resistant to poor water quality and infectious diseases compared to other more sensitive farmed species. These features favored its commercial expansion in recent years. Despite tilapia’s high resistance to pathogens, under some inadequate conditions, mainly those derived from the intensive farming systems or low water quality and/or exposure to environmental contaminants, fish defenses may be compromised. So, infectious disease outbreaks such as the ones derived from parasitic infestations are of major concern (Zago et al. 2014) in farmed tilapia.

To control parasitic diseases two different approaches are commonly used in aquaculture: i) the use of a number of regulated pesticides (Burridge et al. 2010); ii) feeding farmed fish with certain stimulants of the innate immunity, such as β-glucans (Vetvicka et al. 2013; Meena et al. 2013). With respect to the first one, organophosphates are the most used chemicals for parasite control in many fish farms (Tojo and Santamarina 1998). However, if they are delivered indiscriminately, in addition to damaging the environment, it can affect the immune system of fish and thus the overall health, and reduce their productivity (Burridge et al. 2010). In general, bioaccumulation of pesticides may lead to genotoxic, cytotoxic impacts and even mortality in fish (El-Murr et al. 2019). Many pesticides impair the antioxidant system, thus prompting to oxidative stress, which in turn disrupts innate immune responses and produces tissue alterations (Ali et al. 2020). The oxidative stress has been implicated as a possible cause for the non-specific toxicity of organophosphorus compounds, although a variety of enzymatic reactions (oxidation, reduction, hydrolysis, or isomerization) can help to detoxify organophosphorus compounds (Altuntas et al. 2003; Feng et al. 2008). Regarding immunocompetence, oxidative stress limits the production of relevant immune mediators in fish (Biller-Takahashi et al. 2015).

Among the recommended organophosphates, trichlorfon (dimethyl 2,2,2, trichloro-1-hydroxymethyl phosphonate) has been used to eliminate or control a variety of parasites in farmed fish species (Guimarães et al. 2007; Paulsen 2020). Trichlorfon has been associated with immunotoxicity and hematotoxicity in fish, such as common carp (*Cyprinus carpio*) (Chandrasekara and Pathiratne 2005; Woo et al. 2018). Also, trichlorfon is a widely used insecticide and acaricide for the control of various pests in farmlands homes, ornamental plant farms, and against parasites of domestic animals (Lopes et al. 2006; Coelho et al. 2011).

As for the second approach for protection against parasites, immunostimulants focus fish immune defense against these pathogens mainly reinforcing innate immunity, in which the microbicidal activities of macrophages and granulocytes play major roles (Iliev et al. 2005). In this sense, dietary feed supplementation with β-glucans is a recommended procedure to control parasitic infestations in farmed fish (Vetvicka et al. 2013). β-glucans are polysaccharides naturally occurring in the cell walls of some plants, fungi, mushrooms, and bacteria which have immunostimulant properties and are beneficial fed supplements for fish health (Falco et al. 2012; Meena et al. 2013). The use of β-glucans in diets has been shown to promote the stimulation of phagocytosis, the respiratory burst, and production of reactive oxidative species (ROS), and cytokine production in fish macrophages (Meena et al. 2013; Rodrigues et al. 2020).

Moreover, several studies demonstrate that feeding with diets supplemented with β-glucan protects fish from damage caused by pesticides due to its antioxidative potential (El-Murr et al. 2019; Dawood et al. 2020). Although these studies demonstrate the protective role of β-glucans, there is scarce information about the mechanisms that mediate their beneficial effects on innate immunity of immunocompromised fish as a result of the exposure to organophosphates.

To establish the toxicity of aquatic pollutants such as trichlorforn, not only in vivo studies have been performed but also an increasing number of in vitro studies are being conducted using cell lines and primary cell cultures derived from fish organs (Fent 2001; Y. Wang et al. 2011), and also to in vitro analysis of the innate immune responses to immunostimulants (Fierro-Castro et al. 2012, 2013).

The aim of this work is to establish how whether the temporary supply of diets with b-glucan may compensate the effects of water leaching with trichlorfon during the fumigation season in the field, particularly on the effects on the innate immune system of the fish. In this work, we used an in vitro approach to assess the effects of the in vitro exposure to trichlorfon of macrophages isolated from the head kidney (HK macrophages) of tilapia that were fed on a β-glucan-supplemented diet or from tilapia fed on a control (β-glucan-free) diet. We report here the results from in vitro assays investigating the cytotoxicity of trichlorfon on isolated HK macrophages, and then, using non-lethal concentrations of the pesticide, the effects on the intracellular ROS production, the microbicidal capacity, and the expression of relevant immune genes in the HK macrophages after exposure to trichlorfon or to bacterial LPS or to the co-exposure to LPS plus trichlorfon.

## Materials and methods

### Animals

This study was carried out at the Laboratory of Aquaculture of the School of Animal Sciences and Agronomy of the São Paulo State University (FCAT/UNESP) in Dracena, São Paulo, Brazil. Tilapias (*O. niloticus*, Linnaeus, 1758) of 150–200 g were acquired from a local fish farm (Santa Fé do Sul region, São Paulo).

Fish were preserved in recirculating freshwater tanks, at 27 °C and under a 12 h light/12 h dark photoperiod. The fish were kept for 20 days in the facilities to acclimatize and were fed to satiety twice a day with a commercial diet β-glucan free (around 3% of live weight per day with Supra Acqua Line juvenil 42% crude protein, 2.5 mm, Alisul alimentos, Maringá, São Paulo, Brazil).

After acclimatization time, fish were distributed in 2 treatment groups: fish fed on a control β-glucan free diet (control fish) and fish fed on a β-glucan supplemented diet with 0.1% of β-glucan, beta 1,3/1,6 glucan (MacroGard, Bioring**)**, during 15 days. The diets were formulated and prepared at the Fishing Institute of São José do Rio Preto—SP and the fish were fed twice a day until satiation. The composition of these diets is shown in Table 1. Each group consisted of 7 fish per tank, and of 4 tanks per treatment.

**Table 1:** Composition of the experimental diets

Ingredient (g/kg)	Control	β-glucan
Soybean meal	34.2	34.2
Viscera flour	18.0	18.0
Corn	17.5	17.5
Wheat bran	9.00	9.00
Rice crackers	8.00	8.00
Meat and bone meal	6.68	6.68
Protenose	3.44	3.44
Dicalcium phosphate	0.75	0.75
Soy oil	1.00	1.00
Premix^a^	0.50	0.50
Antifungal compound	0.20	0.20
Salt	0.30	0.30
DL-methionine	0.17	0.17
Antioxidant^b^	0.05	0.05
Limestone	0.17	
β-glucan^c^ (60% activity)		0.17
Total	100	100
Proximate composition (%)		
Protein	34.0	34.0
Starch	27.0	27.0
Lipid	6.13	6.13
Ash	8.87	8.87
Crude fiber	3.27	3.27

^a^ Enrichment per kilogram of feed: vit. A: 12,000 UI; vit. D3: 3000 UI; vit. E: 150.00 mg; vit. B1: 20.00 mg; vit. B2: 20.00 mg; vit. B6: 17.50 mg; vit. B12: 40.00 mg; vit. C: 300.00 mg; vit. K: 15.00 mg; folic acid: 6.00 mg; pantothenic acid: 50.00 mg; biotin: 1.00 mg; copper: 17.50 mg; iron: 100.00 mg; iodine: 0.80 mg; manganese: 50.00 mg; niacina: 100.00 mg; zinc: 120.00 mg; colin: 500.00 mg; selenium: 0.50 mg; cobalt: 0.40 mg

^b^ Product contains natural antioxidants, designed to protect animal feed from oxidative deterioration and preserve its freshness. Contains tocopherol (ins 307), soybean lecitin (ins 322), rosemary oil extract, sunflower oil allergic: contains soy derivatives, gluten-free

^c^ β-glucan MacroGard® Biorigin, São Paulo, Brazil

Water quality parameters, temperature 27 ± 0.2 °C, dissolved oxygen 5.3 ± 0.7 mg L^−1^, and pH 7.5 ± 0.3 were monitored daily. The experimental procedures were approved by the Institutional Animal Care Committee of the Sao Paulo State University (CEUA, protocol number 10/2019.R1) and performed in accordance with the guidelines of the Brazilian Council on Animal Care (Colégio Brasileiro de Experimentação Animal, COBEA).

### HK macrophage isolation and culture conditions

Animals were sacrificed by over-anesthetization with tricaine methane sulfonate (MS-222) at a concentration of 200 mg/L. To obtain the macrophages, fish were first bled by puncture in the caudal vein and the HK was removed. The cell culture of HK-derived macrophages was achieved according to the method of Secombes (Secombes 1990) and described by Fierro-Castro et al. (2012). A rich suspension of macrophages was obtained and adjusted to 2 × 10^7^ viable cells mL^−1^ with RPMI medium containing 0.1% FCS, 50 mg mL^−1^ gentamicin, and 2 mg mL^−1^ amphotericin B. The resultant cell suspensions were seeded in 96-well plates (100 µL per well of the suspensions) for the cytotoxicity, respiratory burst, and bactericidal assays or in six-well plates for the gene expression study, and incubated overnight at 28 °C. Purified plastic-adherent macrophages were cultured in fresh RPMI 1640 medium containing 5% FBS, 50 mg mL^−1^ gentamicin, and 2 mg mL^−1^ amphotericin B for 24 h before use.

### Cytotoxicity assay

The cytotoxicity of trichlorfon on HK macrophages was determined by MTT (3-[4,5-dimethylthiazol-2-yl]-2,5-diphenyltetrazolium bromide) reduction assay (Sigma-Aldrich). The cytotoxic activity analyzed with MTT is based on the cellular uptake of MTT and its subsequent reduction to MTT formazan in the mitochondria of viable cells. HK macrophages were exposed to different concentrations of trichlorfon (Masoten®, Bayer, São Paulo, SP, Brasil) (50–2000 µg mL^−1^) and cultured for 24 h. Cell viability was tested by adding 10 µL of MTT (5 mg mL^−1^ in PBS) and incubating in the dark at 28 °C for 2 h. Intracellular formazan crystals were solubilized by adding 100 µL of 100% DMSO and absorbance readings at 570 nm were made with a microplate reader (PerkinElmer, Victor3). Control wells had the same volume of medium without trichlorfon. All experiments were run in triplicate and independently repeated at least three times. Percent viability was calculated as the ratio of absorbance between the test samples and the control: % viability = (Abs sample/Abs control) × 100.

### In vitro exposure to trichlorfon and LPS

HK macrophage cultures (2 × 10^6^ cells per 96-well) were exposed to trichlorfon or to LPS from *Salmonella typhimurium* (Sigma-Aldrich) or to a mixture of LPS plus trichlorfon. Two trichlorfon concentrations (100 and 500 µg mL^−1^) were chosen, as determined from the cytotoxicity assays, so that the lower dose would maintain a cell viability of 95% or higher, and the higher one did not reduce it below 50%. A single LPS concentration of 50 µg mL^−1^ was used, as it has been shown to elicit an inflammatory response in HK macrophages (Fierro-Castro et al. 2012; Liu et al. 2016; Chen et al. 2021). Wells containing unexposed HK macrophages to LPS or trichlorfon were used as controls. After adding the different treatments to the macrophage cultures, the plates were incubated for 24 h under the cell culture conditions described above. The experiments were carried out in triplicate and independently repeated three times.

### Reactive oxygen species (ROS) production

Intracellular ROS production of the macrophages culture exposed to trichlorfon was examined by nitroblue tetrazolium (NBT) reduction assay (Chung and Secombes 1988). In brief, HK macrophages were washed twice with HBSS and 100 µL per well of RPMI medium plus 1 mg mL^−1^ of NBT containing either trichlorfon (100 or 500 µg mL^−1^) or LPS (50 µg mL^−1^) or a mix of trichlorfon and LPS, at the indicated concentrations, was added to the wells. Control wells containing unexposed HK macrophages were established for each plate.

The plates were incubated in the dark at 28 °C for 1 h, and the cells were fixed in methanol (Merck) for 15 min, and then washed with 70% methanol. The obtained formazan product was solubilized by mixing 120 µL of 2 M KOH and 140 µL of DMSO. The absorbance for each sample solution was measured at 620 nm using a microplate reader. The results were expressed as the stimulation index of ROS production calculated as.

$$\mathrm{ROS}\;\mathrm{production}=\left[\left(\mathrm{sample}-\mathrm{blank}\right)/\left(\mathrm{control}-\mathrm{blank}\right)\right]-1$$

The experiments were repeated two times and the results for each test represent the mean of three fish per replicate.

### Bactericidal assay

The bactericidal activity of HK macrophages against the gram-positive coccus *Streptococcus agalactiae* was evaluated after exposure to LPS, trichlorfon, and mixtures of both, at the concentrations and conditions showed above. For this, *S. agalactiae* was cultured in brain heart infusion broth (BHI, cat # 53286, Sigma Aldrich) for 18 h at 27 °C. The bacterial broth was washed by centrifugation at 200 × g for 10 min in Hank’s balanced salt solution without phenol (HBSS, cat. no. 55037C, Sigma Aldrich) (pH 7.4) several times and finally the bacterial solution was adjusted to an optical density of 0. 5 at 600 nm in this salt solution. A bacterial plate count was performed to later infect the HK macrophages at a density of 120 bacteria per macrophage.

After the incubation of the HK macrophages with the different products (LPS, trichlorfon or mixtures of both) as indicated above for 1 h, 20 μL of the bacterial suspension was added to each well and then the plates were centrifuged at 150 × g for 5 min, and incubated at 27 °C for 1 h. After this, the macrophages were lysed by addition of 40 mL/well 0.5% Triton X-100 (cat # T9284, Sigma) solution. Control well containing unexposed cells was established for each plate.

Viable bacteria present in the wells were quantified by the MTT reduction colorimetric method, which is based upon the measurement of the amount of water-insoluble, dark blue MTT-formazan resulting from reduction activity of MTT by mitochondrial dehydrogenases of living bacteria. For this, 20 μL of a solution of 5 mg mL^−1^ of MTT (thiazolyl blue tetrazolium bromide, Sigma Aldrich) in PBS (phosphate buffered saline) was added per well, and the plates were incubated for 1 h. The plates were then read at 620 nm, and the bacterial concentrations were calculated by comparing the absorbance (ABS) obtained for each well to wells without bacteria (blank) and wells with bacterial but not macrophages (positive control, 100% viable bacteria). The percentage of bacterial killing was calculated for each well using the formula = [(positive control − blank) − (sample − blank) × 100 ÷ positive control].

### Gene transcription analysis

After 24 h of exposure to trichlorfon and LPS, total RNA from HK macrophages culture was extracted using TRI reagent (Sigma-Aldrich). Quality and concentration of total RNA were measured spectrophotometrically using a NanoDrop Reader (Thermo Fisher Scientific, USA). A portion of the RNA sample was used to check its integrity by electrophoresis on a 1% agarose gel. One microgram of RNA was used for cDNA synthesis by iScriptTM cDNA Synthesis Kit (Bio-Rad, USA). The resultant cDNA was stored at − 20 °C until use.

The SYBR green method was used with an iQ5 iCycler thermocycler (Bio-Rad) for real-time PCR amplification. Reactions were prepared in 384-well plates according to the manufacturer’s procedures. The genes studied were pro-inflammatory cytokines, interleukin 1 beta (*il-1β*), tumor necrosis factor (*tnfα*) and interleukin 6 (*il-6*), prostaglandin-endoperoxide synthase 2, or cyclooxygenase 2 (*cox2*), a gene involved in signaling inflammatory processes that induces the production of prostaglandins and thromboxane during inflammation, interferon gamma (*ifnγ*), an important factor in the activation of classical macrophages, and the complement component c3b (*c3b*) gene, an important protein during the inflammatory response and the phagocytosis process. The GenBank identification and primer sequences are shown in Table 2. PCR cycling conditions were 95 °C for 10 s, 40 cycles of 95 °C for 30 s, and 60 °C for 30 s, followed by dissociation curve analysis (melting curve). All amplification reactions were run in triplicate *n* = 6. The relative expression ratios of target gene were calculated using the comparative threshold cycle (Ct) method (Pfaffl 2001). The expression of the target genes was normalized using the combination of two reference genes, β-actin and elongation factor-1α-ef1α.

**Table 2:** Sequence of specific primers used for amplification of mRNA transcripts by RT-qPCR of the genes for β-actin, elongation factor-1α, TNFα, IL-1β, IL-6, COX2, C3b, and INFγ

Gene	Fw/Rv sequence (5′–3′)	Pb	Ta	Accession no
Reference gene—house keeping				
*β-actin*	F: AGCCTTCCTTCCTTGGTATGGAAT R: TGTTGGCGTACAGGTCCTTACG	**102**	60 °C	KJ126772
*ef1α*	F: ATCAAGAAGATCGGCTACAACCCT R: ATCCCTTGAACCAGCTCATCTTGT	**109**	60 °C	AB075952.1
Immune-related gene				
*tnfα*	F: CAGAAGCACTAAAGGCGAAGAACA R: TTCTAGATGGATGGCTGCCTTG	**98**	60 °C	NM_001279533/AY428948.1
*il1β*	F: TGGTGACTCTCCTGGTCTGA R: GCACAACTTTATCGGCTTCCA	**86**	60 °C	XM_005457887.3
*il6*	F: ACAGAGGAGGCGGAGATG R: GCAGTGCTTCGGGATAGAG	**165**	60 °C	XM_019350387
*cox2*	F: TGCTGAAAGAGGTCCACCCATACT R: CACTGAGATGCTGCACGTAGTC	**117**	60°	XM_003445052
*c3b*	F: GGTGTGGATGCACCTGAGAA R: GGGAAATCGGTACTTGGCCT	**163**	60 °C	XM_013274267.2
*ifn-γ*	F: TGACCACATCGTTCAGAGCA R: GGCGACCTTTAGCCTTTGT	**128**	58 °C	NM_001287402.1

### Statistical analyses

Data were statistically analyzed using SPSS version 16 statistical software (SPSS Inc, IBM, Chicago, USA). A one-way ANOVA was applied using Tukey’s test for multiple comparisons. Statistical significance was assumed when *P* < 0.01 (**) or *P* < 0.05 (*).

## Results

### Trichlorfon cytotoxicity

**Fig. 1:**
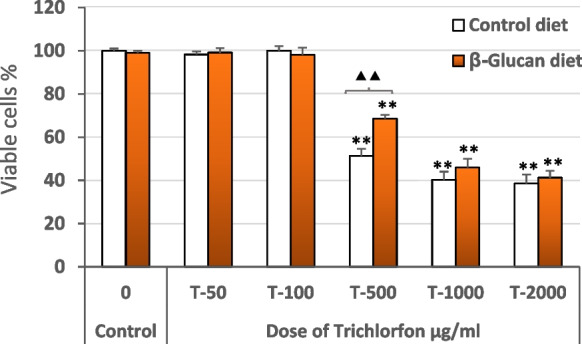
Cytotoxicity of trichlorfon on HK macrophages: viability of tilapia HK macrophages from control (β-glucan free diet) fish (□) and from β-glucan-fish (  ) after exposure to different concentrations of trichlorfon for 24 h. Results are expressed as mean values of triplicate independent experiments (*n* = 6) ± SD. Asterisks indicate significant differences from control cultures (no treatment with trichlorfon) at *P* < 0.05 (*) or *P* < 0.01 (**), while triangles show significant differences at *P* < 0.05 (▲) or *P* < 0.01 (▲▲) between HK macrophages obtained from control fish or from β-glucan-fish

Results from the cytotoxicity assays demonstrated that exposure for 24 h to trichlorfon at concentrations of 100 μg mL^−1^ or lower did not produce any significant cytotoxic effect on HK macrophages, independently of the diet of the fish from which the cells were obtained. At such trichlorfon concentrations, HK macrophages from control fish (β-glucan free diet) and from β-glucan-fish retained a viability higher than 90%, with no significant difference from the viability of the unexposed HM macrophages cultures (Fig. 1).

At concentrations higher than 500 μg mL^−1^, trichlorfon exposure significantly reduced the viability of HK macrophages obtained from the two treatment groups (Fig. 1).

The only difference was observed at the 500 μg mL^−1^ dose, at which the viability of HK macrophages from β-glucan- fish was significantly higher than those obtained from control fish (Fig. 1).

### Reactive oxygen species (ROS) production

**Fig. 2:**
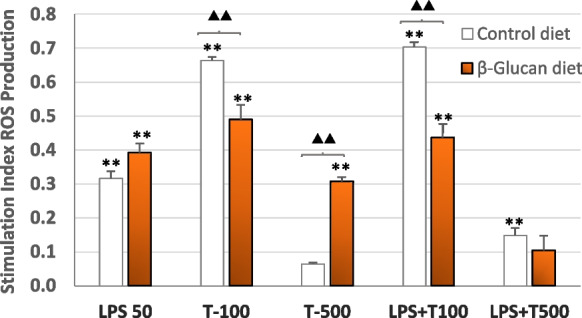
Intracellular ROS production in tilapia HK macrophages from control (β-glucan free diet) fish (□) and from β-glucan-fish (  ) after exposure for 1 h to LPS (50 µg mL^−1^), trichlorfon (100 or 500 µg mL^−1^), or the showed mixtures of both. Results are expressed as the stimulation index (see the “Material and methods” section). Bars represented the mean values of triplicate independent experiments (*n* = 6) ± SD. Asterisks indicate significant differences from the respective unexposed control cultures at *P* < 0.05 (*) or *P* < 0.01 (**), while triangles show significant differences at *P* < 0.05 (▲) or *P* < 0.01 (▲▲), between HK macrophages obtained from control fish or from β-glucan-fish

ROS production was significantly stimulated after exposure for 1 h to LPS (50 μg mL^−1^) and to 100 μg mL^−1^ of trichlorfon in HK macrophages from control fish and β-glucan-fish, but there was a decrease of ROS production at the 500 μg mL^−1^ of trichlorfon, which was more patent for the HK macrophages from control fish (Fig. 2).

Co-exposure to LPS and of 100 μg mL^−1^ of trichlorfon stimulated ROS production in a similar pattern to that observed for LPS alone, but there was also a marked decrease for the highest trichlorfon concentration, at which a significant stimulation was observed only for the HK macrophages from control fish (Fig. 2).

Significant differences between HK macrophages from control fish and from β-glucan-fish were observed for exposures to the two doses of trichlorfon and for the mix of LPS and 100 μg mL^−1^ of trichlorfon (Fig. 2).

### Bactericidal activity in trichlorfon and LPS-treated cells and influence of β-glucan

**Fig. 3:**
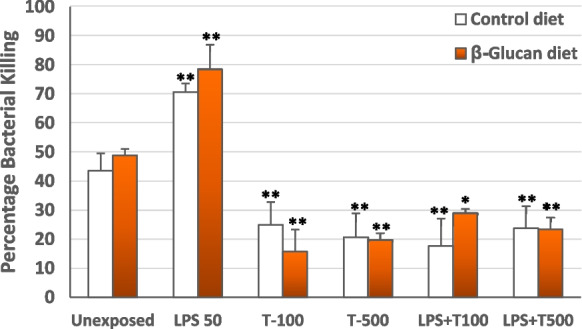
Killing capacity of HK macrophages against *S. agalactiae* from control (β-glucan free diet) fish (□) and from β-glucan-fish (  ) after exposure for 1 h to LPS (50 µg mL^−1^), trichlorfon (100 or 500 µg mL^−1^), or the showed mixtures of both. Results are expressed as percentage of bacterial killing over the positive control wells containing only bacteria. Bars represented the mean values of triplicate independent experiments (*n* = 6) ± SD. Asterisks indicate significant differences from the respective control unexposed HK macrophages at *P* < 0.05 (*) or *P* < 0.01 (**)

Exposure to LPS increased the killing capacity of HK macrophages over that of the control (unexposed) cells, while the exposure to trichlorfon alone or the co-exposure to LPS plus trichlorfon resulted in patent decrease of the bactericidal capacity over that of the controls (Fig. 3).

For any of the experimental conditions of exposure to LPS or trichlorfon or to the mix of the two, there were no significant differences in the killing capacity of HK macrophages from control fish and from β-glucan-fish (Fig. 3).

### Expression of immune-related genes

**Fig. 4:**
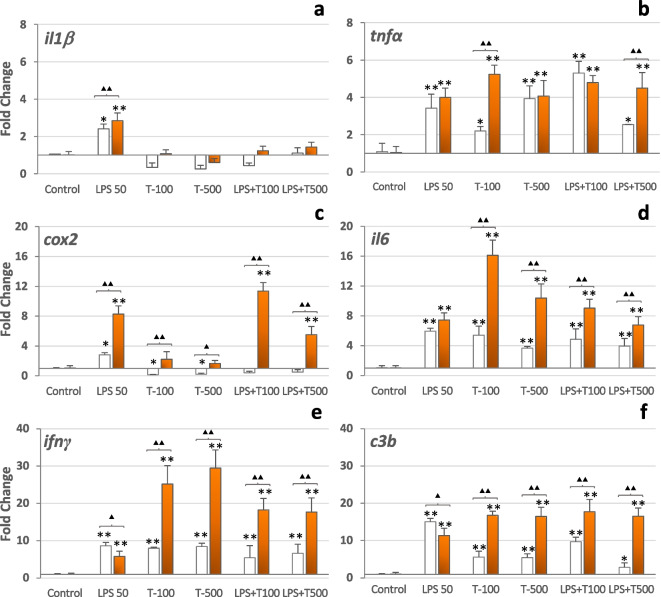
Fold-changes in the relative expression of IL-1β (*il1β*), TNF-α (*tnfα*), IL-6 (*il6*), COX2 (*cox2*), INFγ (*ifnγ*), and complement component c3b (*c3b*) genes in tilapia HK macrophages from control (β-glucan-free diet) fish (□) and from β-glucan-fish (  ) after exposure for 24 h to LPS (50 µg mL^−1^), trichlorfon (100 or 500 µg mL^−1^), or the showed mixtures of both. Bars represent the mean ± SD of relative mRNA expression as fold-changes relative to reference housekeeping genes (*n* = 6 biological replicates). Significant differences are as follows: * vs. control (*P < 0.05 or **P < 0.01); ▲ differences between HK macrophages from control fish or from β-glucan-fish (▲: *P* < 0.05 or ▲▲: *P* < 0.01)

Exposure to LPS, trichlorfon, or mixtures of the two products for 24 h modulated the expression of the analyzed immune-related genes in the HK macrophages from control fish and from β-glucan-fish (Fig. 4). LPS enhanced the expression of all genes in the HK macrophages from the two treatment fish groups against the basal levels found in the respective non-exposed HK macrophages, but there were significant differences between them for some genes. HK macrophages from β-glucan-fish showed significant higher fold changes in the expression of *il1β* (Fig. 4a) and *cox2* (Fig. 4c) genes, while significant decreases occurred in them for the expression of *ifnγ* (Fig. 4e) and *c3b* (Fig. 4f) genes. Also, although the statistical analysis did not detect significant differences, LPS-stimulated HK macrophages from β-glucan-fish showed higher expression levels of the proinflammatory cytokines *tnfα* (Fig. 4b) and *il6* (Fig. 4d) genes than those from control fish.

Exposure of the HK macrophages from the two treatment fish groups to any dose of trichlorfon abrogated the expression of the *il1β* gene (Fig. 4a) and reduced the expression of the *cox2* gen (Fig. 4c), but stimulated the expression of the other genes. Except for the *tnfα* gene at the 500 µg mL^−1^ trichlorfon dose, exposure to this pesticide elicited significant higher increases in the expression of the cytokine *tnfα* (Fig. 4b), *il6* (Fig. 4d), and *ifnγ* (Fig. 4e) genes, of the *cox2* (Fig. 4c) and of the *c3b* (Fig. 4f) genes in HK macrophages from β-glucan-fish than in HK macrophages from control fish. Also, depending on the concentration, trichlorfon-exposed HK macrophages from β-glucan-fish showed higher expression levels than LPS-stimulated HK macrophages from any treatment fish group for the *tnfα* (100 µg mL^−1^, Fig. 3b), *il6* (100 and 500 µg mL^−1^, Fig. 4d) and *ifnγ* (100 and 500 µg mL^−1^, Fig. 4e) genes, but not for the *cox2* (Fig. 4c) and *c3b* genes (Fig. 4f).

The effects of the exposure of the HK macrophages to the mixtures of LPS and the two doses of trichlorfon on the expression of the immune-related genes were similar to those observed after the exposure to trichlorfon alone, except for the *cox2* gene, the expression of which was rescued in the HK macrophages from β-glucan-fish but not on those from control fish (Fig. 4c).

## Discussion

Pesticides, as trichlorfon, have been routinely used to control parasitic infestations in agroindustries including aquaculture, but when applied incorrectly or excessively, in addition to aquatic environmental pollution (Baldissera et al. 2021), they may be harmful to fish health. Thus, immunotoxicity due to the enhancement of oxidative stress, which involves disordering of the ubiquitin–proteasome system, impaired mitochondrial function, endoplasmic reticulum stress, and autophagy, has been described as a consequence of the treatment with pesticides (Mokarizadeh et al. 2015; Lee and Choi 2020). Also, oxidative stress affects the production of different mediators of the immune responses and the proliferation and differentiation of cells of the immune system, so that fish exposed to pesticides may be immunocompromised (Biller-Takahashi et al. 2015). The main finding of the present study is that β-glucan exerted a protective role against oxidative damage in macrophages, but it cannot reduce the deleterious effects of trichlorfon on the microbicidal capacity. The exposure to trichlorfon stimulates various immune responses, such as the overexpression of immune-related genes and the intracellular production of ROS in HK macrophages from both control and β-glucan-fed fish.

Immunostimulants have been commonly used in aquaculture to potentiate the innate immune responses (Bhattacharjee et al. 2020; Baldissera et al. 2021). It is also known that they can counteract the deleterious effects of oxidative stress due to pesticides (Coelho et al. 2011). Among the different immunostimulants, supplementation of the diet with β-glucans is widely used in tilapia aquaculture to improve health, growth performance, and avoid mortality in this species (Koch et al. 2021).

In this study we used an in vitro approach to analyze the effect of the feeding of farmed tilapia with a feed supplemented with β-glucan, a well-known immunostimulant used in aquaculture (Meena et al. 2013), on some of the innate immune responses and of the production of oxidative species in HK macrophages exposed in vitro to trichlorfon, an organophosphate pesticide. HK macrophages from fish feed on a β-glucan-free diet were used as controls. We choose macrophages as the experimental target in this study, as they are key players of the innate immune and of the inflammatory responses, being crucial effector cells of them, but also having important roles in its elicitation and modulation. A similar experimental approach was used to study the effect of trichlorfon on immune gene expression in whole HK leukocyte cell suspensions from Atlantic salmon, *Salmo salar* by Paulsen (2020).

Firstly, we investigated the cytotoxicity of trichlorfon on isolated HK macrophages, because the in vivo effects of trichlorfon of this pesticide have been studied in various fish species, including tilapia (Guimarães et al. 2007; Chang et al. 2020), showing that high doses of trichlorfon damage several tissues (Venkateswara Rao et al. 2003; Guimarães et al. 2007), but no data is available on the cytotoxic activity of this pesticide on macrophage cultures. In our results, exposure for 24 h to trichlorfon did not reduce the viability of the HK macrophages at concentrations equal or below to 100 μg mL^−1^, while concentrations equal or higher than 500 μg mL^−1^ elicited a significant cytotoxic effect, independently of the source of the cells (control fish or β-glucan fish).

Although several mechanisms may be involved, the oxidative stress response to the pesticide may be a major trigger of the cytotoxicity elicited by trichlorfon on the HK macrophages. In this sense, it is of note that only at the 500 μg mL^−1^ concentration, at which a reduction of around a 50% of the viability occurred, HK macrophages from β-glucan-fish exhibited a small, but significant, higher viability than those from control fish (β-glucan-free diet). Such difference may be explained by the protective role of β-glucans against several toxic effects elicited by pesticides in fish, including those arisen from the oxidative stress caused by exposure to such compounds in tilapia (El-Murr et al. 2019; Abdelhamid et al. 2020; Dawood et al. 2020).

Considering the results from the cytotoxicity assay, we chose the 100 and 500 μg mL^−1^ concentrations of trichlorfon for the other assays, so that either no effect or a moderate effect on the viability of the HK macrophages was caused by the exposure to such concentrations of the pesticide. Then, the intracellular ROS production, the microbicidal capacity, and the expression of relevant immune-related genes were analyzed in the HK macrophages from control fish and from β-glucan fish after exposure to the pesticide. Bacterial LPS, a type of the pathogen-associated molecular patterns (PAMPs) agonist of the TLR4 Toll-like receptor (Iliev et al. 2005), was used as a positive control of the stimulation of the HK macrophages mimicking a pathogen infection. Exposures to mixtures of LPS plus trichlorfon were utilized to examine if LPS can rescue the HK macrophage responses that were altered by trichlorfon exposure.

Regarding intracellular ROS production, exposure to trichlorfon stimulated this response in HK macrophages from control fish and from β-glucan-fish, probably reflecting the activation of the oxidative stress response by trichlorfon, a process that has been described in vivo and in vitro (Hai et al. 1997; Peña-Llopis et al. 2003; Feng et al. 2008). Increases in oxidative stress occurred in Nile tilapia leukocytes after in vivo exposure to 0.5 mg L^−1^ trichlorfon for 96 h (Cardoso et al. 2020), and ROS production has also been described in an established skin tumor cell line from carp (*Cyprinus carpio* L.) after exposure to several pesticides (Ruiz-Leal and George 2004).

Notwithstanding the general activation of ROS production in HK macrophages by trichlorfon exposure, this response was modulated by the dose of the pesticide and, in some cases, it varied depending on the fish group (control fish or β-glucan-fish) from which they were isolated. Thus, exposure to 500 μg mL^−1^ trichlorfon alone or mixed with LPS elicited a marked inhibition of the ROS production, which may be due to the decreased viability of the HK macrophages when exposed to this concentration of trichlorfon. Paradoxically, the cytotoxic effect of this dose of trichlorfon may be provoked by an excessive oxidative stress response of the cells to the pesticide, which is a common response to organophosphorus compounds (Mokarizadeh et al. 2015; Lee and Choi 2020) and that, when exacerbated, may cause oxidative damages to the cells (Ding et al. 2019) or immunosuppression (Biller-Takahashi et al. 2015).

On the other hand, the protective effects of β-glucans against cell damage due to oxidative stress that have been reported (El-Murr et al. 2019; Abdelhamid et al. 2020; Dawood et al. 2020) may explain, in part, the differences we observed in the ROS stimulation assay between HK macrophages from control fish and from β-glucan-fish. Thus, when exposed to the lower (100 μg mL^−1^) concentration of trichlorfon that did not reduce the viability of the cells, either alone or in a mix with LPS, HK macrophages from β-glucan fish showed significant lower ROS stimulation levels than those from control fish. In this case, such reduction of the ROS stimulation could be attributed to the inhibitory effects of the feeding with β-glucans on the ROS production in fish macrophages (Meena et al. 2013), although this effect may depend on the β-glucan type and concentration in the diet (Rodrigues et al. 2020).

Conversely, the higher ROS stimulation of the β-glucan-fish HK macrophages exposed to 500 μg mL^−1^ of trichlorfon could be explained by the higher viability of the HK macrophages isolated from β-glucan-fish when exposed to this trichlorfon concentration, as discussed above. So, in this case, there would be more viable cells able to produce intracellular ROS in the cultures of HK macrophages isolated from β-glucan-treated fish.

Microbicidal activity, which is mediated by ROS production and other cell processes, as phagocytosis and extracellular release of antimicrobial compounds, is a main mechanism of the innate immune system for the defense against pathogens (Biller and Takahashi 2018). In this study we used the in vitro bactericidal assay of the HK macrophages as a measure of its overall microbicidal capacity after the different treatments. In our results, the exposure to trichlorfon clearly abrogated the bacterial killing capacity of HK macrophages from β-glucan fish and from control fish, which was not restored by the co-exposure with LPS. Even the lowest concentration of trichlorfon, which showed no significant impact on the viability of the HK macrophages, drastically reduced the bactericidal capacity, thus indicating that this inhibition would be caused by the alterations elicited by the pesticide on some of the cell processes involved in microbicidal ability of the macrophages. In this sense, as indicated above, disruption of key cell organelles (mitochondria, endoplasmic reticulum) and processes involved in phagocytosis and in the release of microbicidal compounds occur in cells exposed to pesticides (Mokarizadeh et al. 2015; Lee and Choi 2020).

During phagocytosis, leukocytes increase their intracellular oxygen expenditure, producing ROS (Schieber and Chandel 2014). The imbalance between increased ROS production and reduction of the antioxidant system that maintains acceptable oxidative levels can be causative of oxidative stress (Schieber and Chandel 2014; Asima 2016) and the corresponding decrease of cellular functions associated with oxidative damage (Biller and Takahashi 2018).

Also, for any of the in vitro experimental conditions, we did not observe significant differences between the bactericidal activity of HK macrophages from β-glucan-fish or from control fish. It has been described that β-glucan-supplemented feed stimulated the microbicidal activity of macrophages in rainbow trout (Jørgensen et al. 1993) and in rohu (*Labeo rohita*) (Misra 2006), but other studies did not detected stimulation of the expression of bactericidal innate immune genes in *C. carpio* after a 7-week supplementation with β-glucan (Harris et al. 2020). In our results β-glucan supplementation did not exert a significant protection against the deleterious effect of in vitro exposure to trichlorfon on the bacterial killing capacity of the HK macrophages. Therefore, trichlorfon exposure can weaken the host’s defense against microbial diseases as such protection is highly dependent on killing activity of the macrophages even after immunostimulation with β-glucans.

Our findings indicate that the transcriptional mRNA levels of several immune mediators in HK macrophages from Nile tilapia are modulated by the in vitro exposure to trichlorfon and that, except for the *il1β* and *cox2* genes, such changes resembled those elicited by the exposure to LPS, a PAMP that in vitro stimulates the expression of many immune-related genes in HK macrophages (Iliev et al. 2005; Fierro-Castro et al. 2012, 2013). Moreover, except for the *cox2* gene, the co-exposure to LPS and trichlorfon elicited patterns of gene expression modulation that were similar to those observed after exposure to trichlorfon alone. These results are in contrast with those described by Paulsen (2020) using HK macrophages from Atlantic salmon (*Salmo salar*) that were exposed to different concentrations (from 25 to 1 µM) of trichlorfon for 48 h, in which no changes in the expression of several immune-related genes (including *cox-2*, *il-1β*, *tnf-α*, and the INF-inducible *Mx protein*) were observed, possibly due to the low dose of trichlorfon used. There are also other previous conflicting reports on the effects of the in vitro exposure of fish tissues to pesticides on the expression of cytokines genes. For instance, in one study, increases in the expression of *il1β* and *tnfα* genes were described in the intestinal tissue of *C. carpio* after exposure to trichlorfon (Chang et al. 2020), but Wang et al. (2017) reported that murine macrophage exposure to the pyrethroid pesticide bifenthrin decreased the expression of genes for IL-1β, IL-6, and TNF-α.

Less is known about the cellular mechanisms that may mediate such a transcriptional response to trichlorfon exposure. However, it is interesting that this pesticide abrogates the expression of the proinflammatory *il1β* gene, whose product (IL-1) promotes the expression of the inducible *cox2* gene (Wang et al. 2016). Therefore, and although we have no data to explain why the expressions of *il1β* and *cox2* genes were down-regulated, as the products of these genes are crucial for the initiation and regulation of the inflammatory response, it is apparent that exposure to trichlorfon may have important consequences in the elicitation and regulation of the inflammatory responses. In this sense, modulation of Cox-2 gene expression is a complicated process that varies in response to different stimulation conditions and even in different cell types (Wang et al. 2016).

On the other hand, regarding the differences between the gene transcriptional changes of HK macrophages from control diet fish and from β-glucan fish, the responses to the exposure to LPS or to trichlorfon plus LPS recapitulates, partially, those reported in studies in which the expression of immune-related genes was analyzed in β-glucans-fed fish after infection with gram-negative pathogens. In rainbow trout (*Oncorhynchus mykiss*) infected with *Aeromonas hydrophila* (Douxfils et al. 2017) and in carp infected with *Aeromonas salmonicida* (Falco et al. 2012), after feeding with β-glucans, the in vivo responses were organ dependent. Administration of β-glucan has been shown to preserve the expression of immune genes, such as IgM and lysozyme, following pesticide exposure (El-Murr et al. 2019), indicating that β-glucan may alleviate the immunotoxic and antioxidant impact of pesticides such as trichlorfon.

The beneficial immunostimulant effects, including β-glucans, on the innate immune responses appear to be also mediated through the epigenetic mechanisms that characterize the so-called *trained immunity*. This process, derived from encounters with PAMPS, facilitates the transcription and activation of immune-related and of antimicrobial effector genes (Foster et al. 2007). Trained immunity facilitates the development of enhanced, but non-specific, responses of the primed immune cells to different pathogens (Garcia-Valtanen et al. 2017; Netea et al. 2020), so that this effect may explain why HK macrophages from β-glucan-fish generally showed higher microbicidal activity and expression levels of immune-related gene after LPS exposure. But, conversely, such priming effect occurring in the β-glucans-fed fish may also train their immune cells to better resist the effects of the various responses to such PAMPs, including an enhanced/excessive ROS production, thus explaining why in our results HK macrophages from β-glucan-fed fish showed increased viability and decreased ROS production after exposure to trichlorfon.

## Conclusions

In summary, the findings of this study reveal that trichlorfon exposure stimulated several immune responses, including overexpression of immune-related genes and the intracellular ROS production in HK macrophages from control and β-glucan-fish. A prior feeding of β-glucan increased the viability of the cells upon exposure to trichlorfon and decreased ROS production, exerting a protective role against damage in these cells. However, the effect of the β-glucan feeding did not seem to be enough to reduce the deleterious effects of the trichlorfon on the microbicidal capacities of HK macrophages to the exposure to trichlorfon.

### Supplementary Information

Below is the link to the electronic supplementary material.

Supplementary file1 (DOCX 165 KB)

## Data Availability

Any extra data that is required will be provided upon request to the author.
