# Supplementary material for: Assessing the effect of β-glucan diets on innate immune response of tilapia macrophages against trichlorfon exposure: an in vitro study
Source: Fish Physiol Biochem. 2023 Dec 15;50(2):527–41. doi: 10.1007/s10695-023-01283-5 (PMC11021296; doi:10.1007/s10695-023-01283-5)
Supplement: Supplementary file 1 — Supplementary file1 (DOCX 165 KB) [file 10695_2023_1283_MOESM1_ESM.docx]

Supplementary file


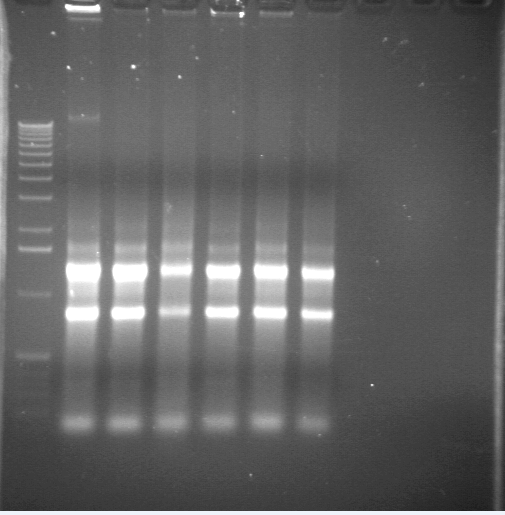


**Fig. A supplementary**: 1% agarose gel electrophoresis of randomly selected RNA extraction samples to check their integrity. 1.5 µg of RNA was loaded in each lane. The left lane contains molecular weight markers. The two upper bands correspond to 28S and 18S ribosomal RNA.
